# Comparison of effectiveness and safety of molnupiravir versus sotrovimab for COVID‐19: A systematic review and meta‐analysis

**DOI:** 10.1002/iid3.1262

**Published:** 2024-04-23

**Authors:** Bahman Amani, Behnam Amani

**Affiliations:** ^1^ Department of Health Management and Economics, School of Public Health Tehran University of Medical Sciences Tehran Iran

**Keywords:** COVID‐19, effectiveness, molnupiravir, safety, SARS‐CoV‐2, sotrovimab

## Abstract

**Background and Aim:**

This systematic review and meta‐analysis aimed to compare the effectiveness and safety of molnupiravir and sotrovimab in the treatment of patients with coronavirus disease 2019 (COVID‐19).

**Methods:**

Cochrane Library, Web of Science, PubMed, medRxiv, and Google Scholar were systematically searched to identify relevant evidence up to December 2023. The risk of bias was assessed using the risk of bias in nonrandomized studies of interventions tool. Data were analyzed using Comprehensive Meta‐Analysis (CMA).

**Results:**

Our search identified and included 13 studies involving 16166 patients. The meta‐analysis revealed a significant difference between the molnupiravir and sotrovimab groups in terms of the mortality rate (odds ratio [OR] = 2.07, 95% confidence interval [CI]: 1.16, 3.70). However, no significant difference was observed between the two groups in terms of hospitalization rate (OR = 0.71, 95% CI: 0.47, 1.06), death or hospitalization rate (OR = 1.51, 95% CI: 0.81, 2.83), and intensive care unit admission (OR = 0.59, 95% CI: 0.07, 4.84). In terms of safety, molnupiravir was associated with a higher incidence of adverse events (OR = 1.67, 95% CI: 1.21, 2.30).

**Conclusion:**

The current findings indicate that sotrovimab may be more effective than molnupiravir in reducing the mortality rate in COVID‐19 patients. However, no statistical difference was observed between the two treatments for other effectiveness outcomes. The certainty of evidence for these findings was rated as low or moderate. Further research is required to provide a better comparison of these interventions in treating COVID‐19 patients.

## Graphical abstract

The present findings may suggest that treatment with sotrovimab is more effective than molnupiravir in reducing mortality rate in COVID‐19 patients. No significant difference was observed between the molnupiravir and sotrovimab in terms of hospitalization rate, death or hospitalization rate, and intensive care unit (ICU) admission. Molnupiravir was associated with higher incidence of adverse events.

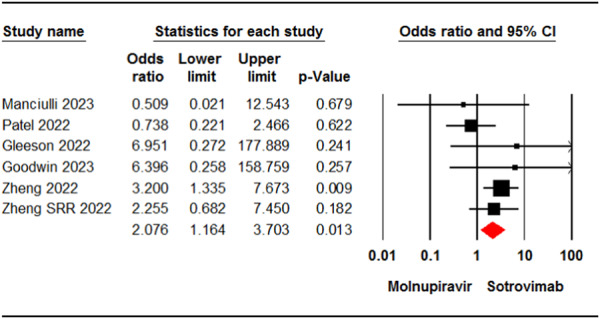

## 1 INTRODUCTION

Considering that widespread vaccination against coronavirus disease 2019 (COVID‐19), caused by the severe acute respiratory syndrome coronavirus 2 (SARS‐CoV‐2) virus, may not provide complete protection for individuals,^1^ it is important to consider other effective therapeutic interventions, especially for populations who are not sufficiently protected by COVID‐19 vaccination.^2^ Recent evidence has demonstrated the potential of certain antiviral agents and monoclonal antibodies in reducing COVID‐19‐related hospitalization and mortality.^3^, ^4^ Real‐world data have highlighted the effectiveness of medications like nirmatrelvir/ritonavir,^5^ remdesivir,^6^ molnupiravir,^7^ and sotrovimab^8^ in patients infected with SARS‐CoV‐2 variants. Those with mild or moderate COVID‐19 who received nirmatrelvir/ritonavir^5^ or remdesivir^6^ treatments showed a decreased risk of death, hospitalization, and severe forms of the disease. Molnupiravir is an oral antiviral medication approved by the US Food and Drug Administration (FDA) for treating high‐risk patients with mild to moderate COVID‐19, aiming to prevent progression to severe disease.^9^ The recommended dosage is a 800 mg oral intake twice daily for 5 days, available as 200‐mg capsules.^10^ Studies have demonstrated the clinical effectiveness of varying doses of molnupiravir in treating COVID‐19 patients.^11^, ^12^, ^13^ Sotrovimab, a human monoclonal antibody, is administered as a single 500 mg IV infusion for patients infected with SARS‐CoV‐2 Delta and Omicron variants.^14^ Studies have shown its effectiveness in reducing mortality and hospitalization rates in COVID‐19 patients and its potential to reduce disease progression and emergency visits in vaccinated individuals.^15^ However, the FDA has suspended its use in regions with a high proportion of the Omicron BA.2 subvariant,^16^ despite real‐world studies demonstrating its effectiveness in patients infected with this subvariant.^17^, ^18^, ^19^ Recent systematic reviews and meta‐analyses have indicated that both molnupiravir and sotrovimab treatments improve clinical outcomes in patients with mild to moderate COVID‐19.^20^, ^21^ Real‐world evidence has revealed diverse findings when comparing the effectiveness of molnupiravir and sotrovimab. Certain studies^7^, ^22^ indicate that sotrovimab outperforms molnupiravir in COVID‐19 patients, while other studies.^23^, ^24^ Currently, both molnupiravir and sotrovimab are approved or authorized for use in countries like the USA, UK, Japan, China, and Australia for COVID‐19 patients.^25^, ^26^ Suggest that molnupiravir is more effective than sotrovimab in treating COVID‐19 patients. As a result, there is a necessity to conduct a systematic review and meta‐analysis to further evaluate the effectiveness of these treatments.

## 2 METHODS

The current research employed the Preferred Reporting Items for Systematic Reviews and Meta‐Analyses (PRISMA) statement (Supporting Information S1: Table 1).^27^ The systematic review and meta‐analysis protocol was registered on PROSPERO with the registration number CRD42023429910.

### 2.1 Literature search

Two researchers independently conducted a comprehensive search using keywords in the Cochrane Library, Web of Science, PubMed, medRxiv, and Google Scholar, aiming to identify relevant evidence up to December 2023. In addition, they scanned the references of systematic reviews and key studies to identify additional records. Keywords included “SARS‐COV‐2,” “COVID‐19,” “molnupiravir,” and “sotrovimab.” No language restrictions were applied. The search strategy for each database is provided in the Supporting Information.

### 2.2 Study selection

The present systematic review and meta‐analysis included studies that met the following criteria: patients with a positive polymerase chain reaction (PCR) COVID‐19 test, treatment with molnupiravir or sotrovimab as monotherapy, and reporting effectiveness and safety outcomes such as mortality rate, hospitalization rate, and adverse events. Studies with irrelevant outcomes, case reports, and those conducted on healthy individuals were excluded.

### 2.3 Risk of bias assessment and quality of evidence

Two researchers independently evaluated the potential for bias in the included studies by utilizing the Risk of Bias in Nonrandomized Studies of Interventions (ROBINS‐I) tool.^28^ This tool covers seven domains, such as confounding, selection bias, measurement and classification biases of interventions, deviations from intended interventions, missing data, outcome measurement biases, and selection of reported results. Additionally, two authors assessed the quality of evidence for each outcome using the Grading of Recommendations, Assessment, Development, and Evaluations (GRADE) tool, which incorporates criteria such as risk of bias, imprecision, inconsistency, indirectness, and publication bias. The evidence was categorized into four levels: very low, low, moderate, and high.

### 2.4 Data extraction

Two researchers independently extracted all necessary data using a standardized extraction form. The extracted information encompassed: (1) study characteristics (including first author, year of publication, study location, and design type); (2) patient details (total sample size, gender, and average age); (3) treatment interventions (sample size, average age, dosage, duration of treatment, COVID‐19 vaccination rate, and extent of comorbidities); (4) effectiveness outcomes (mortality rate, hospitalization rate, death or hospitalization rate, and ICU admission); and (5) safety outcomes (incidence of any adverse events).

### 2.5 Data analysis

The effectiveness and safety of molnupiravir and sotrovimab were compared using Comprehensive Meta‐Analysis (CMA). For analyzing dichotomous variables, the odds ratio (OR) with a 95% confidence interval (CI) was utilized. High heterogeneity was defined as *I* ^2^ > 50% or *p* < .1, in which case a random‐effect model was applied. Conversely, the fixed effects model was employed for studies without high heterogeneity. Subgroup analyses were carried out based on patients' age (<60 years and ≥60 years) and the sample size of studies (<500 and >500). Moreover, sensitivity analyses were conducted in the following manner. Initially, studies with a high risk of bias were excluded from the comparison with the overall effect size. Second, two studies by Zheng et al.^7^, ^22^ utilized OpenSAFELY‐TPP platform data to evaluate the effectiveness of molnupiravir and sotrovimab in COVID‐19 patients. Considering the potential overlap of data between these studies, one of them was excluded, and a subsequent meta‐analysis for the outcomes of interest was performed.

## 3 RESULTS

### 3.1 Search result

**Figure 1:**
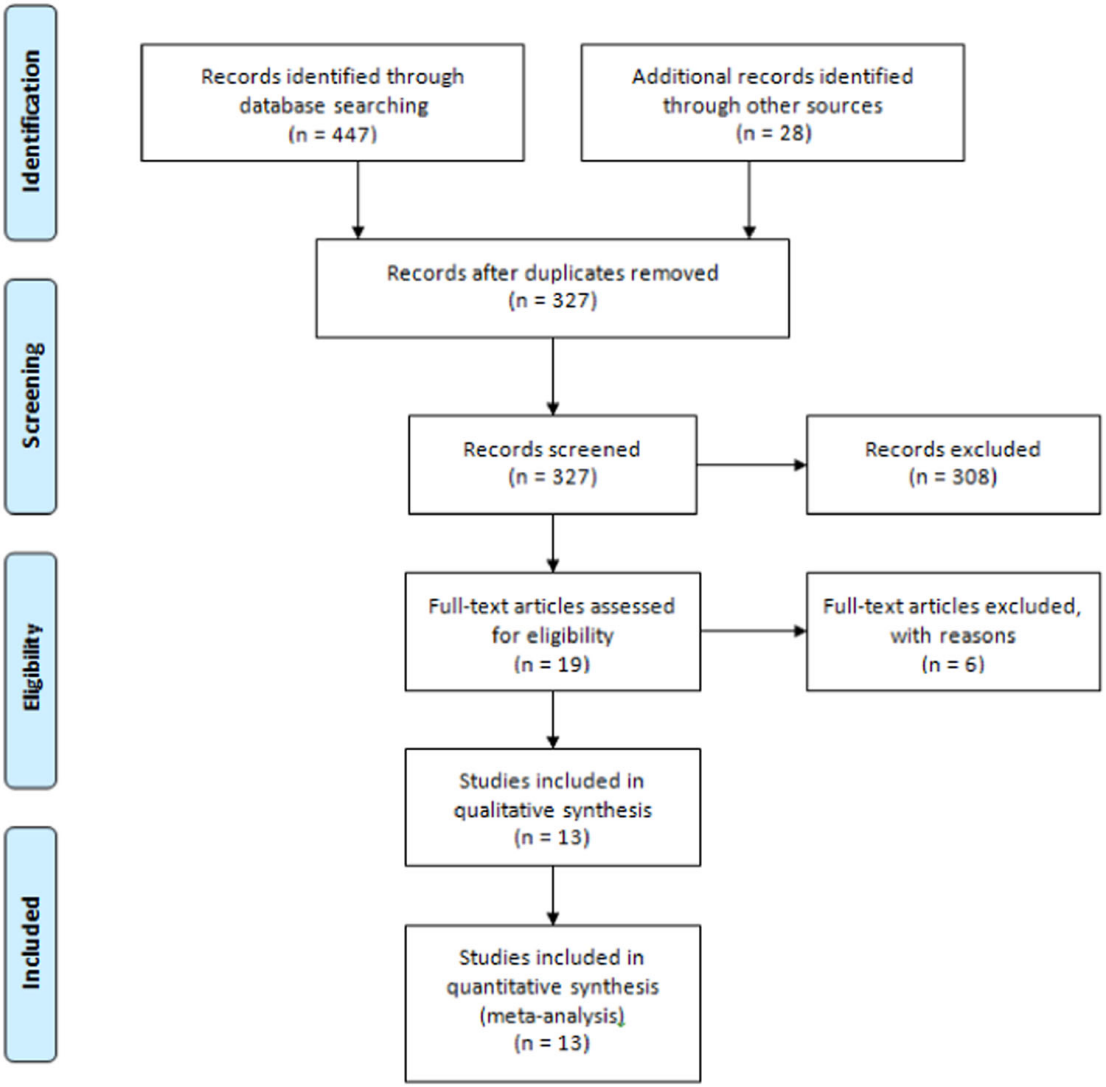
Flow diagram of PRISMA. PRISMA, Preferred Reporting Items for Systematic Reviews and Meta‐Analyses.

Figure 1 depicts the study selection process based on title, abstract, and full‐text review. Following the removal of duplicate records, 327 articles were assessed against the inclusion criteria. Nineteen studies were considered eligible for full‐text review, out of which six were excluded based on the criteria outlined in Figure 1. Ultimately, 13 studies^7^, ^22^, ^23^, ^24^, ^29^, ^30^, ^31^, ^32^, ^33^, ^34^, ^35^, ^36^, ^37^ involving 16,166 patients were included in the systematic review and meta‐analysis. All included studies featured a retrospective design, with most comparing more than two intervention groups. The administered doses were 800 mg twice daily for molnupiravir and a single 500 mg infusion for sotrovimab. The follow‐up duration for the majority of studies was 28 days, and the predominant location of the studies was the UK. The key characteristics of the included studies are outlined in Table 1.

**Table 1:** Characteristics of studies included in the meta‐analysis.

Study	Year	Place	Molnupiravir					Sotrovimab					Follow‐up (days)
			Mean age	*N*	Male	Comorbidity (%)^a^	Vaccination rate (%)^b^	Mean age	*N*	Male	Comorbidity (%)^a^	Vaccination rate (%)^b^	
Cegolon^29^	2023	Italy	66.2	116	62	80.2	81.9	69.8	57	36	87.7^c^	87.7	30
Drysdale^30^	2023	UK	59.6	71	29	NA	90	54.6	492	206	NA	100	28
Evans^31^	2023	UK	56	359	140	59.9	97	54	1079	442	58.6	98.5	28
Gleeson^23^	2022	UK	NA	21	NA	NA	NA	NA	47	NA	NA	NA	97
Goodwin^32^	2023	UK	51	80	50	NA	98.8	52	169	64	NA	98.7	28
Kauer^33^	2023	Austria	61	1788	839	NA	NA	64	420	201	NA	NA	28
Lasagna^34^	2022	Italy	NA	7	5	100	100	NA	3	1	100	100	14
Manciulli^24^	2023	Italy	68.9	205	118	>50	88.3	64.7	314	166	>50	79.6	28
Mazzotta^35^	2022	Italy	68	117	65	>50	93.1	63	202	97	>50	91	30
Patel^36^	2022	UK	53.7	470	253	>50	96.6	58	696	340	>50	95.1	28
Radcliffe^37^	2022	USA	55	49	25	NA	92	57	24	14	NA	88	≤30
Zheng^22^	2022	UK	52.9	2689	1138	NA	97.4	51.7	3331	1340	NA	98.1	28
Zheng‐ UKRR^7^	2022	UK	55.5	515	298	86.8	98.2	56	1852	1039	85.6	98.5	28
Zheng‐SRR^7^	2022	UK	54.7	270	157	NA	100	58.4	723	413	NA	97.8	28

Abbreviations: *N*, number; NA, not applicable.

^a^ Having at least one comorbidity.

^b^ Receipt of ≥1 dose SARS‐CoV‐2 vaccine.

^c^ Having at least three comorbidity.

### 3.2 Risk of bias assessment and quality of evidence

Risk of confounding for most studies was evaluated as moderate. All studies had low risk of Classification of interventions and Missing Data. Deviations from intended interventions and Measurement of outcomes for all studies were moderate. Risk of other domains varied in studies. The detailed results of risk of bias assessment using ROBINS‐I tool are presented in Supporting Information S1: Table 2. Moreover, the assessment of the certainty of evidence for each outcome is presented in Supporting Information S1: Table 3.

**Table 2:** Subgroup and sensitivity analyses for effectiveness outcomes.

Analysis	No. of studies	Sample size	Point estimate (95% CI)	*p* Value	Heterogeneity		
					*χ* ^2^	*p* Value	*I* ^2^ (%)
*Sensitivity analysis*							
Mortality rate (excluding Zheng SSRR)	5	8022	2.02 [1.04, 3.92]	0.03	5.50	0.23	27.32
Death or hospitalization (excluding Zheng UKRR)	4	8794	1.21 [0.58, 2.54]	0.60	9.93	0.01	69.79
Mortality rate (excluding Celegon 2023)	5	8947	1.99 [1.10, 3.59]	0.02	4.97	0.29	19.62
Hospitalization rate (excluding Celegon 2023 and Kauer 2023)	6	2085	0.93 [0.59, 1.48]	0.78	6.12	0.29	18.32
*Subgroup analysis*							
Mortality rate by age							
<60	5	8496	2.17 [1.20, 3.92]	0.01	4.76	0.31	16.02
≥60	1	519	0.50 [0.02, 12.54]	0.67	0.00	1.00	0.00
Hospitalization rate by age							
<60	4	1556	0.89 [0.54, 1.46]	0.65	5.82	0.12	48.51
≥60	3	2518	0.44 [0.21, 0.89]	0.02	3.76	0.15	46.80
Death or hospitalization rate by age							
<60	4	10,818	1.75 [1.30, 2.35]	3.72	0.00	10.30	70.88
≥60	1	343	0.12 [0.00, 2.20]	0.15	0.00	1.00	0.00
Mortality rate by sample size							
<500	2	317	6.66 [0.68, 65.27]	0.10	0.00	0.97	0.00
≥500	4	9015	1.91 [1.05, 3.48]	0.03	4.45	0.21	32.63
Hospitalization rate by sample size							
<500	5	573	1.02 [0.40, 2.56]	0.96	3.88	0.14	48.46
≥500	3	3511	0.65 [0.41, 1.02]	0.06	7.76	0.10	48.49
Death or hospitalization rate by sample size							
<500	1	343	0.12 [0.00, 2.20]	0.00	10.30	0.01	70.88
≥500	4	10,818	1.75 [1.30, 2.35]	0.15	0.00	1.00	0.00

Abbreviations: CI, confidence interval; NA, not applicable; UKRR, UK Renal Registry.

### 3.3 Effectiveness outcomes

#### 3.3.1 Mortality rate

**Figure 2:**
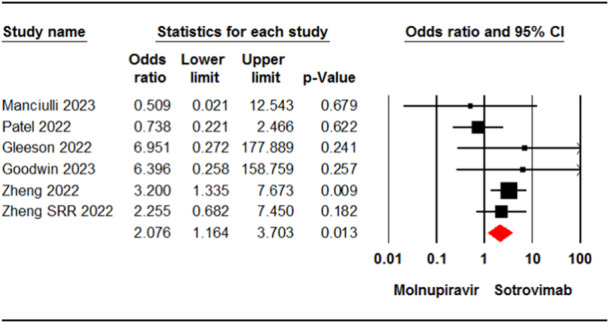
Forest plot of molnupiravir versus sotrovimab for mortality rate. CI, confidence interval.

Six studies^7^, ^22^, ^23^, ^24^, ^32^, ^36^ involving 9015 patients reported cases of death in patients who received molnupiravir or sotrovimab. The pooled estimate of these studies showed a significant difference in mortality rate between the two groups (OR = 2.07, 95% CI: 1.16, 3.70, *p* = .01) (Figure 2). The certainty of evidence for this outcome was rated as moderate.

#### 3.3.2 Hospitalization rate

**Figure 3:**
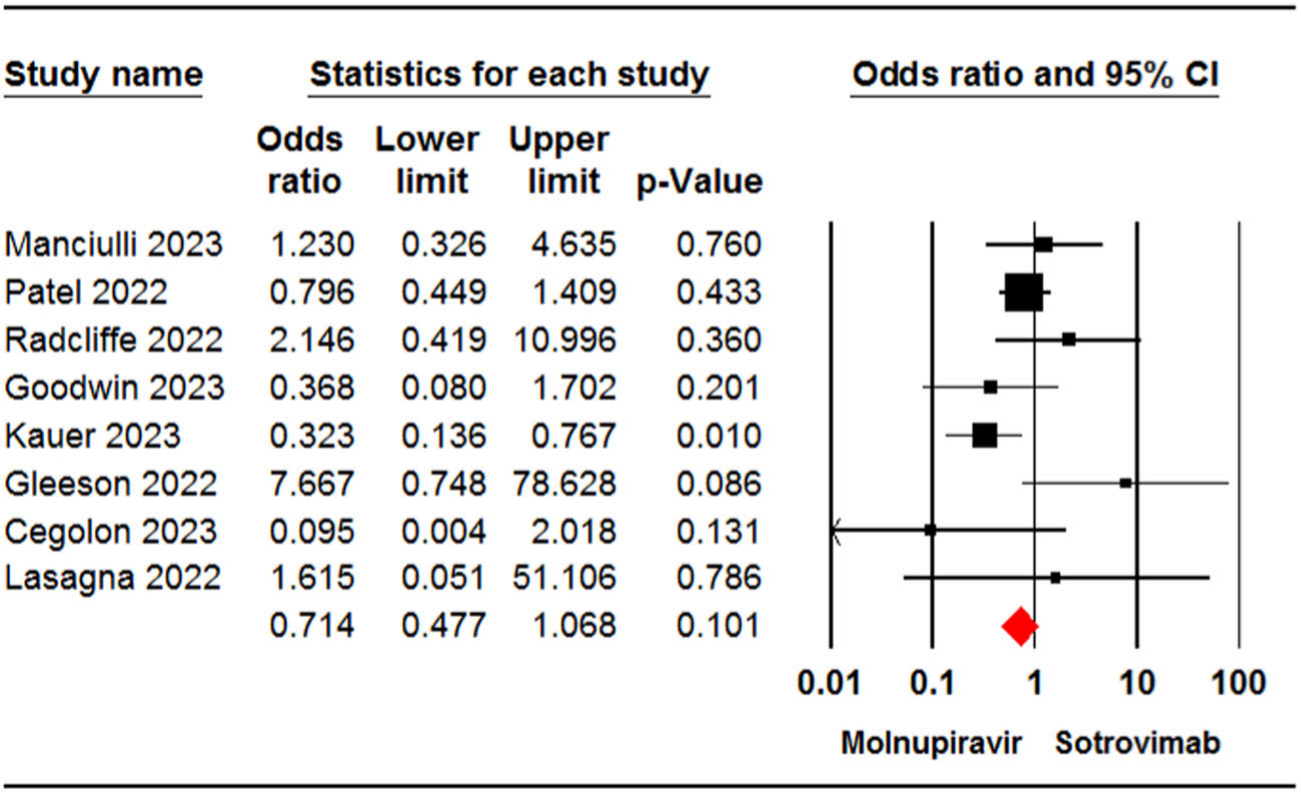
Forest plot of molnupiravir versus sotrovimab for hospitalization rate. CI, confidence interval.

Eight studies^23^, ^24^, ^29^, ^32^, ^33^, ^34^, ^36^, ^37^ involving 4084 patients reported cases of hospital admission for patients receiving molnupiravir or sotrovimab. The meta‐analysis revealed no significant difference in hospitalization rate between the two groups (OR = 0.71, 95% CI: 0.47, 1.06, *p* = .10) (Figure 3). The certainty of evidence for this outcome was rated as moderate.

#### 3.3.3 Death or hospitalization rate

**Figure 4:**
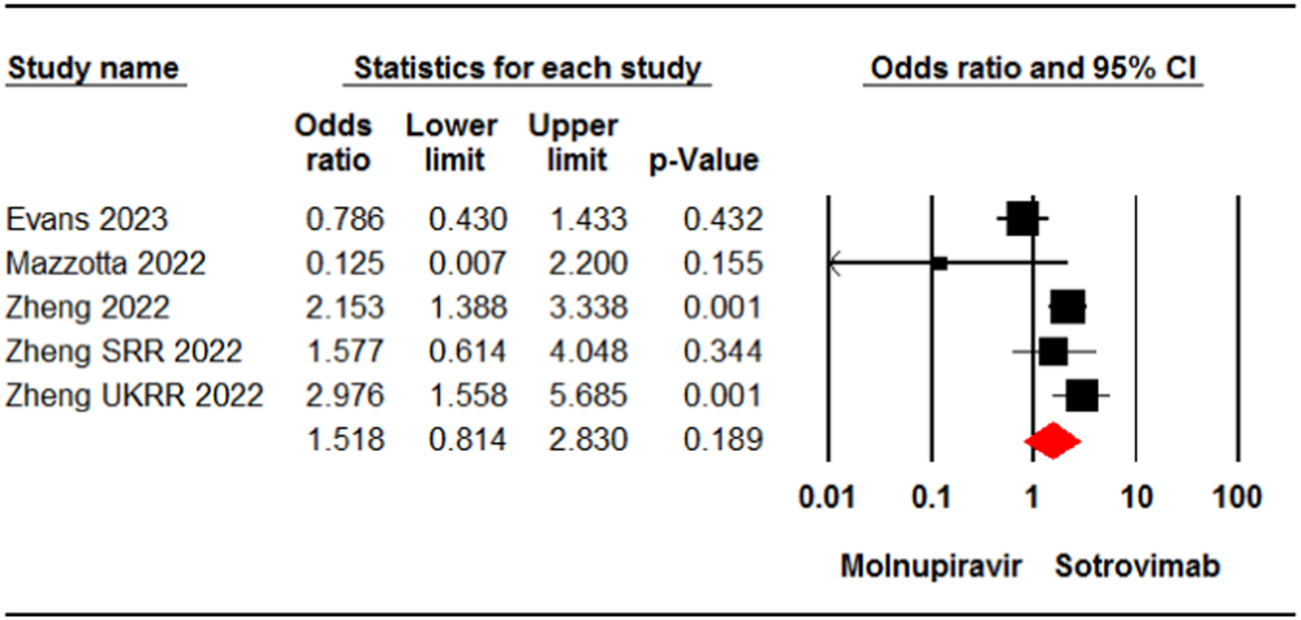
Forest plot of molnupiravir versus sotrovimab for death or hospitalization rate. CI, confidence interval.

Four studies^7^, ^22^, ^31^, ^35^ involving 11,161 patients reported cases of death or hospitalization in patients who received molnupiravir or sotrovimab. The meta‐analysis showed no significant difference between the two groups (OR = 1.51, 95% CI: 0.81, 2.83, *p* = .18) (Figure 4). The certainty of evidence for this outcome was rated as low.

#### 3.3.4 ICU admission

**Figure 5:**
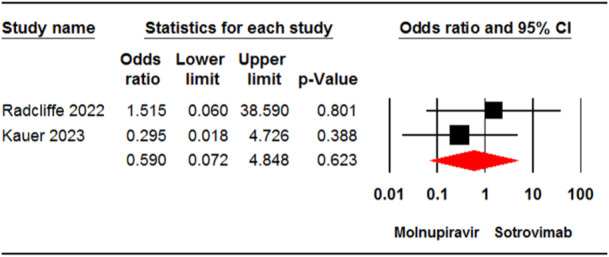
Forest plot of molnupiravir versus sotrovimab for intensive care unit admission. CI, confidence interval.

Two studies^33^, ^37^ involving 1428 patients were included in the meta‐analysis for ICU admission. The analysis showed no significant difference between patients receiving molnupiravir and those who received sotrovimab in terms of admission to the ICU (OR = 0.59, 95% CI: 0.07, 4.84, *p* = .62) (Figure 5). The certainty of evidence for this outcome was rated as low.

### 3.4 Safety outcomes

#### 3.4.1 Any adverse events

**Figure 6:**
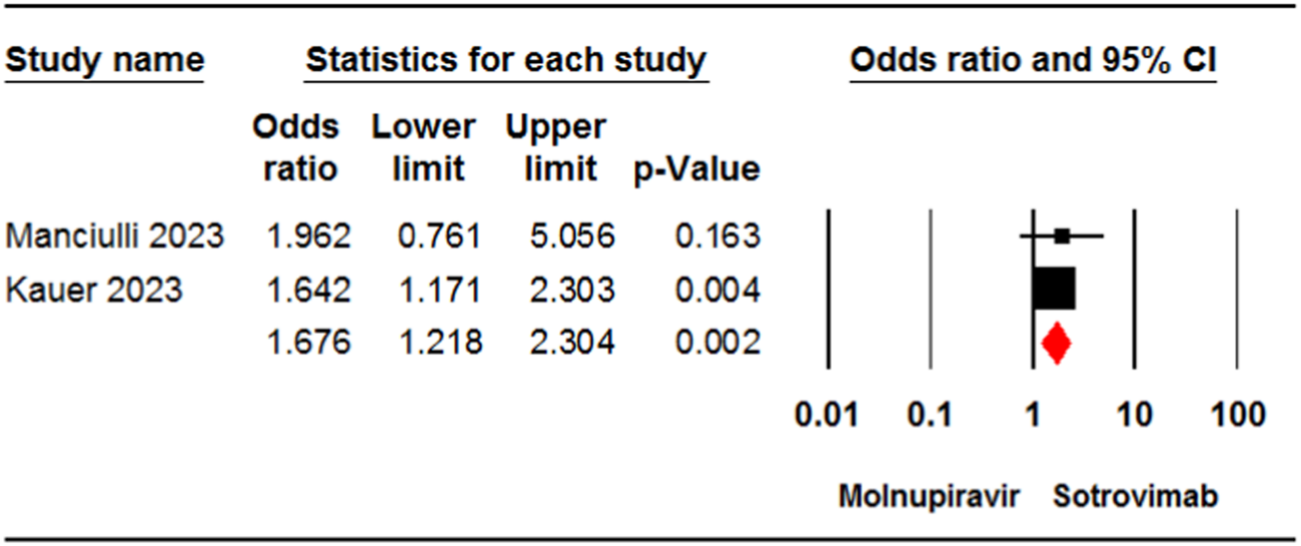
Forest plot of molnupiravir versus sotrovimab for adverse events. CI, confidence interval.

Two studies^24^, ^33^ involving 2177 patients reported the incidence of any adverse events in the molnupiravir and sotrovimab groups. The pooled estimate demonstrated a significant difference between the two treatment groups in terms of the incidence of any adverse events (OR = 1.67, 95% CI: 1.21, 2.30, *p* < .01) (Figure 6). The certainty of evidence for this outcome was rated as low.

### 3.5 Subgroup and sensitivity analyses

Table 2 displays the results of subgroup analysis by the age of patients and the sample size of studies. The sensitivity analysis conducted by excluding one of the studies indicated no significant change compared to the primary analysis for the outcomes of mortality rate (OR = 2.02, 95% CI: 1.04, 3.92, *p* = .03) and death or hospitalization rate (OR = 1.21, 95% CI: 0.58, 2.54, *p* = .60) (Table 2). Furthermore, the pooled estimates were similar to the primary analysis when we excluded studies with a high risk of bias for outcomes of mortality rate (OR = 1.99, 95% CI: 1.10, 3.59, *p* = .02) and hospitalization rate (OR = 0.93, 95% CI: 0.59, 1.48, *p* = .78).

## 4 DISCUSSION

While certain variants of SARS‐CoV‐2 have posed challenges in developing effective treatments for COVID‐19,^38^ recent real‐world data indicate the effectiveness of molnupiravir and sotrovimab in treating patients infected with SARS‐CoV‐2 variants.^20^, ^21^ The aim of this systematic review and meta‐analysis was to compare the effectiveness and safety of molnupiravir versus sotrovimab in COVID‐19 patients.

The current meta‐analysis reveals that treatment with sotrovimab is linked to a significantly reduced risk of mortality in COVID‐19 patients compared to those treated with molnupiravir. This superiority may be attributed to sotrovimab's greater effectiveness in reducing the risk of severe COVID‐19 in patients, as opposed to those treated with molnupiravir.^7^, ^22^ A comparative effectiveness study by Zheng et al.^22^ illustrated that the risk of death in patients infected with Omicron variants was lower in the sotrovimab group compared to the molnupiravir group. Additionally, meta‐analysis of real‐world studies has indicated that sotrovimab could significantly reduce the risk of death in patients infected with SARS‐CoV‐2 Delta or Omicron variants. Furthermore, in vivo and in vitro studies^39^, ^40^, ^41^ studies have demonstrated the effective activity of sotrovimab against certain Omicron SARS‐CoV‐2 variants. However, the administration of sotrovimab in COVID‐19 patients should be carefully considered in light of possible mutations.^42^ On the other hand, the effectiveness of molnupiravir in reducing COVID‐19‐related mortality remains unclear. While some meta‐analyses found no significant benefit in using molnupiravir to reduce COVID‐19‐related mortality,^21^, ^43^, ^44^ others showed it to be effective in reducing the risk of death in COVID‐19 patients.^45^, ^46^ Discrepancies in these findings may be attributed to differences in inclusion criteria and the number of included studies. Current data from studies also support the effectiveness of nirmatrelvir/ritonavir and remdesivir treatments in reducing the risk of death in patients with COVID‐19. Nonetheless, evidence regarding the effectiveness of antiviral and monoclonal treatments in children with COVID‐19 is limited.^47^ Although one study^47^ found no deaths in COVID‐19 children who received treatments of molnupiravir or sotrovimab, further research is needed to establish the effectiveness of these interventions for children with COVID‐19.

The current meta‐analysis findings indicate that both molnupiravir and sotrovimab treatments have similar effects on reducing the hospitalization rate in COVID‐19 patients. Previous evidence from our meta‐analysis supports the idea that COVID‐19 patients treated with sotrovimab were less likely to be hospitalized compared to those not receiving sotrovimab.^20^ However, the results of meta‐analyses on the therapeutic potential of molnupiravir are inconclusive and conflicting. Recent meta‐analyses conducted by Benaicha et al.^45^ and Gao et al.^46^ have shown that molnupiravir significantly reduces the hospitalization rate in COVID‐19 patients, while others have found no significant effect when compared to no treatment with molnupiravir.^43^, ^44^ Two other drugs approved by the FDA, nirmatrelvir/ritonavir and remdesivir, have demonstrated effectiveness in reducing the likelihood of hospitalization in patients who receive these treatments.

According to the current meta‐analysis, there is no significant difference between molnupiravir and sotrovimab in reducing death or hospitalization rates in COVID‐19 patients. Our previously published study demonstrated that sotrovimab significantly reduced the death or hospitalization rate in COVID‐19 patients compared to the control group.^20^ However, a meta‐analysis conducted by Malin et al.^43^ on randomized control trials found no significant difference in reducing death or hospitalization rates between patients who received molnupiravir and those who received placebo/standard of care. In real‐world settings, evidence comparing the effectiveness of molnupiravir and sotrovimab in COVID‐19 patients varies. A real‐world cohort study published by Zheng et al.^22^ showed that sotrovimab is more effective than molnupiravir in reducing death or hospitalization rates in adult patients with COVID‐19. According to their study, the death or hospitalization rate for COVID‐19 patients receiving molnupiravir and sotrovimab were 2.05% and 0.96%, respectively. On the other hand, one study reported no COVID‐19‐related hospitalization or death for patients treated with molnupiravir, while 3.1% of patients who received sotrovimab experienced COVID‐19‐related hospitalization or death.^35^

The current meta‐analysis also indicates that COVID‐19‐related ICU admission was similar in patients treated with molnupiravir or sotrovimab. Real‐world meta‐analyses have demonstrated that sotrovimab infusion was linked to a reduced risk of ICU admission due to COVID‐19 compared to the nonsotrovimab group.^20^ However, evidence from real‐world data presents different findings when comparing these interventions in terms of reducing ICU admission for COVID‐19 patients. Kauer et al.^33^ found a similar rate of ICU admission between nonhospitalized patients at high risk for COVID‐19 who received molnupiravir and sotrovimab. In contrast, a study by Radcliffe et al.^37^ found that none of the solid organ transplant recipients infected with COVID‐19 who received sotrovimab were admitted to the ICU, whereas 2% of the molnupiravir group had ICU admission. Given that only two studies were included in the meta‐analysis to compare the effectiveness of molnupiravir and sotrovimab in reducing ICU admission, the present finding in regard this should be interpreted with caution.

The incidence of adverse events was significantly higher in patients receiving molnupiravir compared to those receiving sotrovimab. Recently published meta‐analyses revealed that there was no significant difference in the incidence of adverse events between the molnupiravir and control groups.^21^, ^44^, ^46^ Additionally, our previous research revealed no significant difference in the incidence of adverse events between different doses of molnupiravir (200, 400, and 800 mg) and placebo in COVID‐19 patients.^48^ This discrepancy can be attributed to the type of control groups in the meta‐analyses. In the aforementioned studies, standard care or placebo was considered as the control group; while in the present meta‐analysis sotrovimab was considered as the comparison. Regarding the safety of sotrovimab, a meta‐analysis of real‐world studies revealed a comparable incidence of adverse events in COVID‐19 patients treated with sotrovimab compared to those untreated with sotrovimab.^20^ The most frequently reported adverse events for COVID‐19 patients taking sotrovimab in studies were nausea, dizziness, headaches, and rash.^24^, ^32^, ^33^ However, Kauer et al.^33^ reported one serious adverse event of atrial fibrillation in a COVID‐19 patient who received sotrovimab. Patients taking molnupiravir experienced adverse events, mostly diarrhea, vomiting, high blood pressure, headaches, and dizziness.^24^, ^33^ Manciulli et al.^24^ reported drug discontinuation in patients taking molnupiravir, while no discontinuation occurred in patients taking sotrovimab. Several cases of drug intolerance were reported in patients taking either molnupiravir or sotrovimab.^24^

Our study is subject to several significant limitations. Firstly, the designs of all studies included in the present meta‐analysis were observational, which are associated with a high risk of bias. Moreover, some studies failed to report key variables that could affect the treatment effect, such as COVID‐19 vaccination status, comorbidity rates, and a history of re‐infection in patients. Furthermore, two studies included in our analysis did not administer treatments within 5 days after the onset of COVID‐19 symptoms, potentially impacting the estimation of treatment effects. Finally, many studies featured more than two intervention groups, which could influence the estimation of treatment effects. Conducting a network meta‐analysis may provide a more robust comparison of these interventions.

## 5 CONCLUSION

The study's findings indicate that during the Omicron prevalent period, molnupiravir and sotrovimab demonstrate similar effectiveness in reducing hospitalization rate, death or hospitalization rate, and ICU admission in COVID‐19 patients. However, sotrovimab is notably superior to molnupiravir in reducing the mortality rate in COVID‐19 patients. In terms of safety, molnupiravir is associated with a higher incidence of adverse events in COVID‐19 patients. Nonetheless, the certainty of evidence supporting these findings is rated as low or moderate. These results can offer valuable insights for healthcare policymakers, clinicians, and researchers regarding the comparison of these interventions in COVID‐19 patients. In the future, research efforts should prioritize the comparison of the effectiveness of these interventions on newly circulating SARS‐COV‐2 variants. This will help in gaining a comprehensive understanding of their possible long‐term impacts on both the SARS‐COV‐2 variants and public health.

## AUTHOR CONTRIBUTIONS

*Conceptualization and project administration*: Bahman Amani and Behnam Amani. *Literature searching*: Behnam Amani and Bahman Amani. *Data extraction and quality assessment*: Bahman Amani and Behnam Amani. *Data analysis*: Bahman Amani and Behnam Amani. *Writing—original draft*: Behnam Amani. *Writing—review and editing*: Bahman Amani and Behnam Amani.

## CONFLICT OF INTEREST STATEMENT

The authors declare no conflict of interest.

## Supporting information

Supporting information.

## Data Availability

Data are available online for the included studies.^7^, ^22^, ^23^, ^24^, ^29^, ^31^, ^32^, ^33^, ^35^, ^36^, ^37^

## References

[1] Calimeri S , Lo Giudice D , Buda A , et al. Role of the 1st booster dose of COVID‐19 vaccine in the protection against the infection: a fundamental public health tool. J Prev Med Hyg. 2022;63(4):520. 10.15167/2421-4248/jpmh2022.63.4.2742 PMC9986990 36891000

[2] Caillard S , Thaunat O . COVID‐19 vaccination in kidney transplant recipients. Nat Rev Nephrol. 2021;17(12):785‐787. 34580488 10.1038/s41581-021-00491-7 PMC8475856

[3] Lin WT , Hung SH , Lai CC , Wang CY , Chen CH . The impact of neutralizing monoclonal antibodies on the outcomes of COVID‐19 outpatients: a systematic review and meta‐analysis of randomized controlled trials. J Med Virol. 2022;94(5):2222‐2229. 35088444 10.1002/jmv.27623 PMC9015482

[4] Wen W , Chen C , Tang J , et al. Efficacy and safety of three new oral antiviral treatment (molnupiravir, fluvoxamine and Paxlovid) for COVID‐19: a meta‐analysis. Ann Med. 2022;54(1):516‐523. 35118917 10.1080/07853890.2022.2034936 PMC8820829

[5] Wong C , Au I , Lau K , Lau E , Cowling BJ , Leung GM . Real‐world effectiveness of early molnupiravir or nirmatrelvir‐ritonavir in hospitalised patients with COVID‐19 without supplemental oxygen requirement on admission during Hong Kong's omicron BA.2 wave: a retrospective cohort study. Lancet Infect Dis. 2022;22(12):1681‐1693. 36029795 10.1016/S1473-3099(22)00507-2 PMC9401976

[6] Ramos‐Rincon J‐M , López‐Carmona M‐D , Cobos‐Palacios L , et al. Remdesivir in very old patients (≥ 80 years) hospitalized with COVID‐19: real world data from the SEMI‐COVID‐19 registry. J Clin Med. 2022;11(13):3769. 35807058 10.3390/jcm11133769 PMC9267524

[7] Zheng B , Campbell J , Carr EJ , et al. Comparative effectiveness of sotrovimab and molnupiravir for preventing severe COVID‐19 outcomes in non‐hospitalised patients on kidney replacement therapy: observational cohort study using the OpenSAFELY‐UKRR linked platform and SRR database. medRxiv . Preprint posted online December 4, 2022. 10.1101/2022.12.02.22283049 PMC10616487 37915915

[8] De Vito A , Colpani A , Poliseno M , et al. What is the efficacy of sotrovimab in reducing disease progression and death in people with COVID‐19 during the omicron era? Answers from a real‐life study. Viruses. 2023;15(8):1757. 37632099 10.3390/v15081757 PMC10458484

[9] Kamal L , Ramadan A , Farraj S , Bahig L , Ezzat S . The pill of recovery; Molnupiravir for treatment of COVID‐19 patients; a systematic review. Saudi Pharm J. 2022;30(5):508‐518. 10.1016/j.jsps.2022.03.002 35287313 PMC8906919

[10] Painter WP , Holman W , Bush JA , et al. Human safety, tolerability, and pharmacokinetics of molnupiravir, a novel broad‐spectrum oral antiviral agent with activity against SARS‐CoV‐2. Antimicrob Agents Chemother. 2021;65(5):02428‐20. 10.1128/aac PMC8092915 33649113

[11] Flisiak R , Zarębska‐Michaluk D , Rogalska M , et al. Real‐world experience with molnupiravir during the period of SARS‐CoV‐2 Omicron variant dominance. Pharmacol Rep. 2022;74(6):1279‐1285. 36001284 10.1007/s43440-022-00408-6 PMC9400562

[12] Kwok WC , Tsoi MF , Leung SHI , et al. Real‐world study on effectiveness of molnupiravir and Nirmatrelvir–Ritonavir in unvaccinated patients with chronic respiratory diseases with confirmed SARS‐CoV‐2 infection managed in out‐patient setting. Viruses. 2023;15(3):610. 36992319 10.3390/v15030610 PMC10055981

[13] Tiseo G , Barbieri C , Galfo V , et al. Efficacy and safety of nirmatrelvir/ritonavir, molnupiravir, and remdesivir in a real‐world cohort of outpatients with COVID‐19 at high risk of progression: the PISA outpatient clinic experience. Infect Dis Ther. 2023;12(1):257‐271. 36441485 10.1007/s40121-022-00729-2 PMC9707131

[14] Cheng MM , Reyes C , Satram S , et al. Real‐world effectiveness of sotrovimab for the early treatment of COVID‐19 during SARS‐CoV‐2 Delta and Omicron waves in the USA. Infect Dis Ther. 2023;12:607‐621. 36629998 10.1007/s40121-022-00755-0 PMC9832411

[15] Nene RV , Santodomingo MA , Balog B , et al. Use of sotrovimab in vaccinated versus unvaccinated COVID‐19 patients in a resource‐limited emergency department during the omicron surge. J Am Coll Emerg Physicians Open. 2023;4(3):e12958. 37188260 10.1002/emp2.12958 PMC10175722

[16] Food and Drug Administration. Drug safety and availability . 2022. https://www.fda.gov/drugs/drug-safety-and-availability/fda-updates-sotrovimab-emergency-use-authorization

[17] Behzad A , Mohamed A , Ali A , Niinuma S , Butler AE , Alqahtani M . Real world effectiveness of sotrovimab in preventing COVID‐19‐related hospitalisation or death in patients infected with Omicron BA.2. J Infect Public Health. 2023;17(2):315‐320. 38160562 10.1016/j.jiph.2023.11.029

[18] Martin‐Blondel G , Marcelin A‐G , Soulié C , et al. Sotrovimab to prevent severe COVID‐19 in high‐risk patients infected with Omicron BA.2. J Infect. 2022;85(4):e104‐e108. 35803386 10.1016/j.jinf.2022.06.033 PMC9254651

[19] Miyashita N , Nakamori Y , Ogata M , et al. Clinical efficacy of the neutralizing antibody therapy sotrovimab in patients with SARS‐CoV‐2 omicron BA.1 and BA.2 subvariant infections. Viruses. 2023;15(6):1300. 37376600 10.3390/v15061300 PMC10302949

[20] Amani B , Amani B . Efficacy and safety of sotrovimab in patients with COVID‐19: a rapid review and meta‐analysis. Rev Med Virol. 2022;32(6):e2402. 36226323 10.1002/rmv.2402 PMC9874927

[21] Huang PY , Liu TH , Wu JY , Tsai YW , Lai CC . Clinical efficacy and safety of molnupiravir for nonhospitalized and hospitalized patients with COVID‐19: a systematic review and meta‐analysis of randomized control trials. J Med Virol. 2023;95(3):e28621. 36846901 10.1002/jmv.28621

[22] Zheng B , Green ACA , Tazare J , et al. Comparative effectiveness of sotrovimab and molnupiravir for prevention of severe covid‐19 outcomes in patients in the community: observational cohort study with the OpenSAFELY platform. BMJ. 2022;379:e071932. 10.1136/bmj-2022-071932 36384890 PMC9667468

[23] Gleeson S , Martin P , Thomson T , et al. Kidney transplant recipients and omicron: outcomes, effect of vaccines and the efficacy and safety of novel treatments. medRxiv . Preprint posted online May 3, 2022. 10.1101/2022.05.03.22274524

[24] Manciulli T , Spinicci M , Rossetti B , et al. Safety and efficacy of outpatient treatments for COVID‐19: real‐life data from a regionwide cohort of high‐risk patients in Tuscany, Italy (the FEDERATE Cohort). Viruses. 2023;15(2):438. 36851654 10.3390/v15020438 PMC9967010

[25] Maas BM , Strizki J , Miller RR , et al. Molnupiravir: mechanism of action, clinical, and translational science. Clin Transl Sci. 2024;17(2):e13732. 38593352 10.1111/cts.13732 PMC10851176

[26] Twohig KA , Nyberg T , Zaidi A , et al. Hospital admission and emergency care attendance risk for SARS‐CoV‐2 delta (B.1.617.2) compared with alpha (B.1.1.7) variants of concern: a cohort study. Lancet Infect Dis. 2022;22(1):35‐42. 34461056 10.1016/S1473-3099(21)00475-8 PMC8397301

[27] Page MJ , McKenzie JE , Bossuyt PM , et al. The PRISMA 2020 statement: an updated guideline for reporting systematic reviews. Int J Surg. 2021;88:105906. 33789826 10.1016/j.ijsu.2021.105906

[28] Sterne JA , Hernán MA , Reeves BC , et al. ROBINS‐I: a tool for assessing risk of bias in non‐randomised studies of interventions. BMJ. 2016;355:i4919. 27733354 10.1136/bmj.i4919 PMC5062054

[29] Cegolon L , Pol R , Simonetti O , Larese Filon F , Luzzati R . Molnupiravir, Nirmatrelvir/Ritonavir, or Sotrovimab for high‐risk COVID‐19 patients infected by the omicron variant: hospitalization, mortality, and time until negative swab test in real life. Pharmaceuticals. 2023;16(5):721. 37242504 10.3390/ph16050721 PMC10221734

[30] Drysdale M , Tibble H , Patel V , et al. Characteristics and outcomes of patients with COVID‐19 at high risk of disease progression receiving sotrovimab, oral antivirals or no treatment in Scotland. medRxiv . Preprint posted online June 9, 2023. 10.1101/2023.06.09.23291195 PMC11225175 38965495

[31] Evans A , Qi C , Adebayo JO , et al. Real‐world effectiveness of molnupiravir, nirmatrelvir‐ritonavir, and sotrovimab on preventing hospital admission among higher‐risk patients with COVID‐19 in Wales: a retrospective cohort study. J Infect. 2023;86(4):352‐360. 36773891 10.1016/j.jinf.2023.02.012 PMC9911979

[32] Goodwin AT , Thompson JS , Hall IP . Evaluation of outpatient treatment for non‐hospitalised patients with COVID‐19: the experience of a regional centre in the UK. PLoS One. 2023;18(3):e0281915. 36920896 10.1371/journal.pone.0281915 PMC10016683

[33] Kauer V , Totschnig D , Augustin M , Karolyi M , Zoufaly A , Naegeli M . Efficacy and tolerability of Sotrovimab Molnupiravir and Nirmatrelvir/Ritonavir for non‐hospitalized patients at high risk for COVID‐19: a retrospective, single‐center analysis. Preprint posted online April 10, 2023. 10.21203/rs.3.rs-2786240.v1

[34] Lasagna A , Cassaniti I , Lilleri D , et al. Effectiveness of the available early therapies in reducing severe COVID‐19 in non‐hospitalized patients with solid tumors on active treatment. Front Med. 2022;9:1036473. 10.3389/fmed.2022.1036473 PMC9643502 36388947

[35] Mazzotta V , Cozzi Lepri A , Colavita F , et al. Viral load decrease in SARS‐CoV‐2 BA.1 and BA.2 Omicron sublineages infection after treatment with monoclonal antibodies and direct antiviral agents. J Med Virol. 2023;95(1):e28186. 36184918 10.1002/jmv.28186 PMC9539310

[36] Patel V , Yarwood MJ , Levick B , et al. Characteristics and outcomes of patients with COVID‐19 at high‐risk of disease progression receiving sotrovimab, oral antivirals or no treatment in England. *medRxiv*. Preprint posted online November 29, 2022. 10.1101/2022.11.28.22282808 38975862

[37] Radcliffe C , Palacios CF , Azar MM , Cohen E , Malinis M . Real‐world experience with available, outpatient COVID‐19 therapies in solid organ transplant recipients during the omicron surge. Am J Transplant. 2022;22(10):2458‐2463. 10.1111/ajt.17098 35583664 PMC9348251

[38] Fernandes Q , Inchakalody VP , Merhi M , et al. Emerging COVID‐19 variants and their impact on SARS‐CoV‐2 diagnosis, therapeutics and vaccines. Ann Med. 2022;54(1):524‐540. 35132910 10.1080/07853890.2022.2031274 PMC8843115

[39] Driouich J‐S , Bernadin O , Touret F , de Lamballerie X , Nougairède A . Activity of Sotrovimab against BQ.1.1 and XBB.1 Omicron sublineages in a hamster model. Antiviral Res. 2023;215:105638. 37207822 10.1016/j.antiviral.2023.105638 PMC10191698

[40] Hérate C , Marlin R , Touret F , et al. Sotrovimab retains activity against SARS‐CoV‐2 Omicron variant BQ.1.1 in a non‐human primate model. Heliyon. 2023;9:e16664. 37287613 10.1016/j.heliyon.2023.e16664 PMC10228175

[41] Touret F , Baronti C , Bouzidi HS , de Lamballerie X . In vitro evaluation of therapeutic antibodies against a SARS‐CoV‐2 Omicron B.1.1.529 isolate. Sci Rep. 2022;12(1):4683. 35304531 10.1038/s41598-022-08559-5 PMC8931583

[42] Uraki R , Kiso M , Imai M , et al. Therapeutic efficacy of monoclonal antibodies and antivirals against SARS‐CoV‐2 Omicron BA.1 in Syrian hamsters. Nat Microbiol. 2022;7(8):1252‐1258. 35705860 10.1038/s41564-022-01170-4 PMC12990156

[43] Malin JJ , Weibel S , Gruell H , Kreuzberger N , Stegemann M , Skoetz N . Efficacy and safety of molnupiravir for the treatment of SARS‐CoV‐2 infection: a systematic review and meta‐analysis. J Antimicrob Chemother. 2023;78(7):1586‐1598. 37170886 10.1093/jac/dkad132 PMC10320168

[44] Tian F , Feng Q , Chen Z . Efficacy and safety of molnupiravir treatment for COVID‐19: a systematic review and meta‐analysis of randomized controlled trials. Int J Antimicro Agents. 2023;62:106870. 10.1016/j.ijantimicag.2023.106870 PMC10214763 37245600

[45] Benaicha K , Khenhrani RR , Veer M , et al. Efficacy of molnupiravir for the treatment of mild or moderate COVID‐19 in adults: a meta‐analysis. Cureus. 2023;15(5):e38586. 37284377 10.7759/cureus.38586 PMC10239651

[46] Gao Y , Liu M , Li Z , Xu J , Zhang J , Tian J . Molnupiravir for treatment of adults with mild or moderate COVID‐19: a systematic review and meta‐analysis of randomised controlled trials. Clin Microbiol Infect. 2023;29(8):979‐999. 37084941 10.1016/j.cmi.2023.04.014 PMC10116122

[47] Minotti C , Mengato D , De Pieri M , et al. Early treatments of fragile children with COVID‐19—results of CLEVER (Children COVID Early Treatment), a retrospective, observational study. Viruses. 2023;15(1):192. 36680232 10.3390/v15010192 PMC9867507

[48] Amani B , Zareei S , Amani B . Rapid review and meta‐analysis of adverse events associated with molnupiravir in patients with COVID‐19. Br J Clin Pharmacol. 2022;88(10):4403‐4411. 35762036 10.1111/bcp.15449 PMC9349444

